# [Retracted] Tumor promoter role of miR-647 in gastric cancer via repression of TP73

**DOI:** 10.3892/mmr.2026.13980

**Published:** 2026-08-03

**Authors:** Xiangqian Zhang, Min Zhang, Guifeng Wang, Ye Tian, Xiaolong He

Mol Med Rep 18: 3744–3750, 2018; DOI: 10.3892/mmr.2018.9358

Following the publication of the above paper, it was drawn to the Editor's attention by a concerned reader that certain of the flow cytometric plots featured in Fig. 4 on p. 3748 were strikingly similar to plots that had appeared in a pair of articles written by different authors at different research institutes that had already been submitted for publication/published in the journal *Medical Science Monitor*, and a further paper with which the above paper shared flow cytometric data was identified as having been submitted to the journal *Oncology Letters*.

Owing to the fact that the contentious data in the above article had already been published prior to its submission to *Molecular Medicine Reports*, the Editor has decided that this paper should be retracted from the Journal. The authors were asked for an explanation to account for these concerns, but the Editorial Office did not receive a reply. The Editor apologizes to the readership for any inconvenience caused.

